# Cutaneous Metastasis in a Treated Case of Cervical Cancer With Extraordinary Response to Chemotherapy: A Case Report of a Rare Event and Review of the Literature

**DOI:** 10.7759/cureus.35083

**Published:** 2023-02-16

**Authors:** Bikash Ranjan Mahapatra, Anupam Muraleedharan, Avinash Badajena, Saroj Kumar Das Majumdar, Nehla Haroon K M

**Affiliations:** 1 Radiation Oncology, Utkal Institute of Medical Sciences, Bhubaneswar, Bhubaneswar, IND; 2 Radiation Oncology, All India Institute of Medical Sciences, Bhubaneswar, Bhubaneswar, IND

**Keywords:** 18f-fdg pet-ct, bevacizumab, radiotherapy, cutaneous metastasis, carcinoma cervix

## Abstract

Cervical cancer usually metastasizes to the lung, liver, bone, and brain. Metastasis to the skin from cervical cancer is relatively uncommon. The management options are systemic therapy, palliative radiotherapy, or best supportive care. Here, we report the case of a female patient with cervical cancer, stage IIB, who received radical treatment with radiotherapy and chemotherapy and later presented with disseminated skin nodules. She was treated with combination chemotherapy (nano-dispersible paclitaxel and carboplatin), bevacizumab, and a bone-stabilizing agent (zoledronic acid). There was a complete metabolic response to the therapy. There was also a dramatic improvement in the general condition of the patient. Skin metastasis in cervical cancer often presents as non-tender skin nodules. A biopsy is mandatory to establish the diagnosis. There are no specific guidelines about management. The intention of management is palliative. The combination of chemotherapy and bevacizumab produces substantial clinical improvement.

## Introduction

Cervical cancer is more prevalent in developing and underdeveloped parts of the world. This is due to the lack of screening, low socioeconomic status, and lack of access to healthcare and vaccination against the human papillomavirus (HPV). Worldwide, cervical cancer is the fourth highest contributor to all female malignancies [1]. In India, it is the second most common malignancy in females after breast cancer, with an annual incidence of 0.12 million [2]. The management of cervical cancer depends on the stage of the disease. Early-stage tumors are managed either with surgery or brachytherapy.

On the other hand, definitive chemoradiotherapy followed by brachytherapy is the treatment of choice. The five-year survival rate in localized cervical cancer is 92%, which drops drastically to 17% for metastatic disease [3]. The lung, liver, bone, and brain are the common sites of distant metastasis from cervical cancer. Skin as a location of metastasis is quite unusual, and the prognosis is invariably poor. We report a case of cervical cancer with skin metastasis that developed after one year of completion of local treatment. The dramatic response to the biological response modifier makes this case unique and, thus, a worthy contribution to existing literature.

## Case presentation

**Figure 1:**
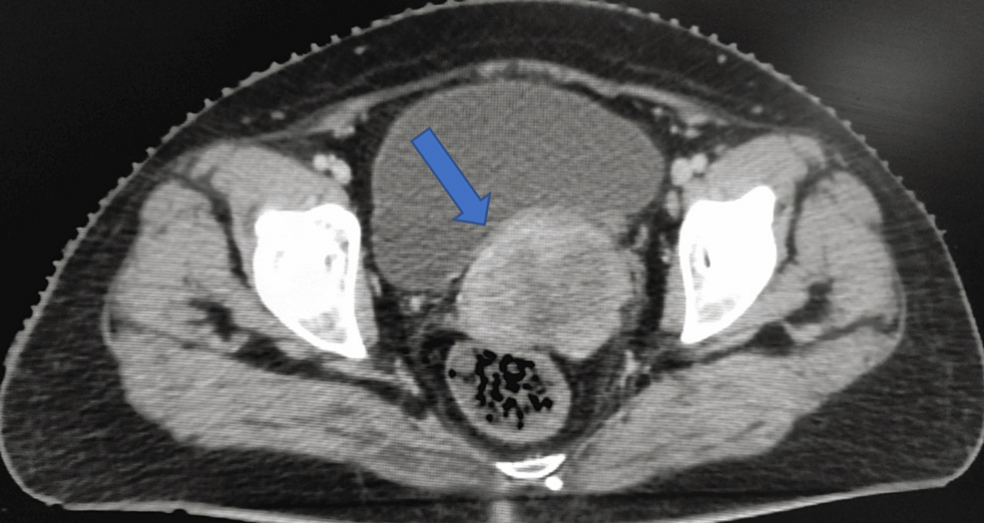
Axial section of the CECT of the pelvis showing the 6 cm × 7.5 cm × 9 cm lesion involving the cervix. CECT: contrast-enhanced computed tomography

A 55-year-old postmenopausal female with an obstetric score of para 2, living 2 presented with the chief complaint of bleeding per vaginum for three months. This was not associated with fever, trauma, white discharge per vaginum, bleeding per rectum, abdominal pain, or backache. On general examination, the patient was anemic. Per vaginal and per speculum examination, there was a 4 cm × 4 cm friable, exophytic growth involving the whole cervix. Per rectal examination, bilateral medial parametrium was indurated (left side > right side). The pelvic walls and rectal mucosa were free. A provisional diagnosis of cervical cancer was made. For confirmation of the diagnosis, a biopsy was taken from the cervical growth, and a pelvic examination was done under spinal anesthesia, which revealed the same findings. The histopathology was moderately differentiated squamous cell carcinoma (grade 2). A contrast-enhanced computed tomography (CECT) scan was done for staging workup. It showed a 6 cm × 7.5 cm × 9 cm heterogeneously enhancing lesion involving the lower part of the uterus and cervix and extending into the upper two-thirds of the vaginal canal (Figure 1).

**Figure 2:**
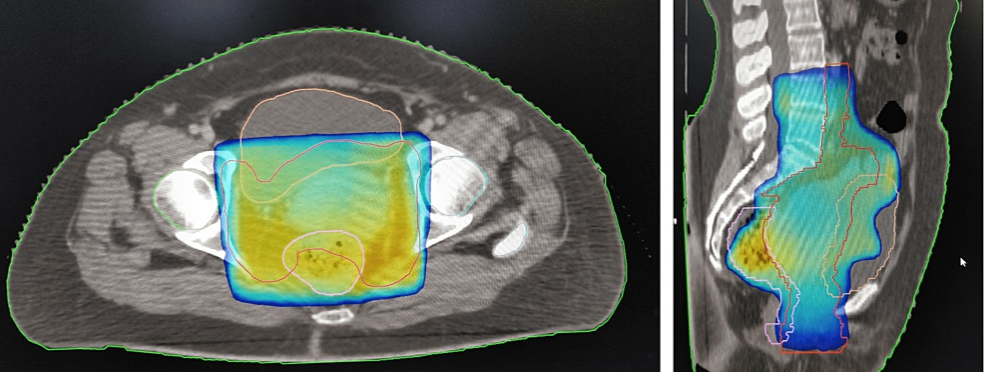
3DCRT plan of EBRT in axial and sagittal sections with color wash corresponding to 95% isodose coverage. 3DCRT: three-dimensional conformal radiotherapy, EBRT: external beam radiotherapy

So, the patient was finally diagnosed with carcinoma of the cervix International Federation of Gynaecology and Obstetrics (FIGO) stage IIB. A complete hemogram, kidney function tests, liver function tests, audiometry, and echocardiography were done. All tests were within normal limits. The patient was planned for concurrent chemoradiotherapy (CCRT) followed by intracavitary brachytherapy (ICBT). She received external beam radiotherapy (EBRT) 50.4 Gray in 28 fractions over five weeks with a three-dimensional conformal radiotherapy technique (3DCRT) using mixed beam energy (6 and 10 megavolt photons) in Versa HD linear accelerator (Elekta, Stockholm, Sweden) (Figure 2).

She also received six cycles of weekly concurrent injection cisplatin 40 mg/m^2^. The patient tolerated the EBRT with Radiation Therapy Oncology Group grade 1 skin and lower gastrointestinal toxicity.

**Figure 3:**
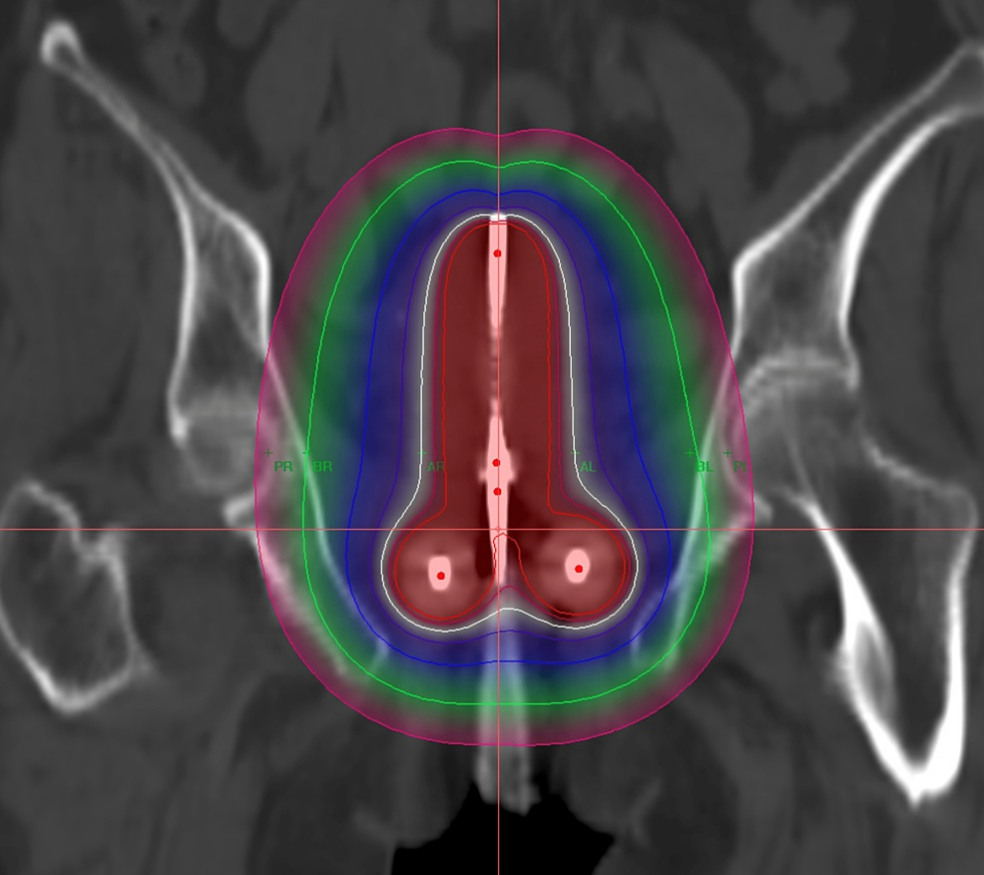
Color wash of the ICBT plan in the coronal section. The white isodose line shows the 7 Gray volume. The dark red line is 110%, the light blue line is 50%, and the green line is 30% isodose lines of the prescribed dose. ICBT: intracavitary brachytherapy

She then was subjected to three sittings of high-dose-rate intracavitary brachytherapy (ICBT), 7 Gray per sitting, over three weeks under spinal anesthesia (Figure 3).

The total treatment duration was eight weeks.

After three months of completion of treatment, the patient was evaluated clinically and radiologically (contrast-enhanced magnetic resonance imaging (CEMRI) of the pelvis and CECT of the thorax and abdomen) and was disease-free. After 15 months of completion of treatment, the patient developed multiple non-tender subcutaneous swellings over the right palm, forehead, and right arm, along with a dry cough and diffuse body aches. Upon presentation, the patient used a wheelchair with an Eastern Cooperative Oncology Group (ECOG) performance status of 3.

**Figure 4:**
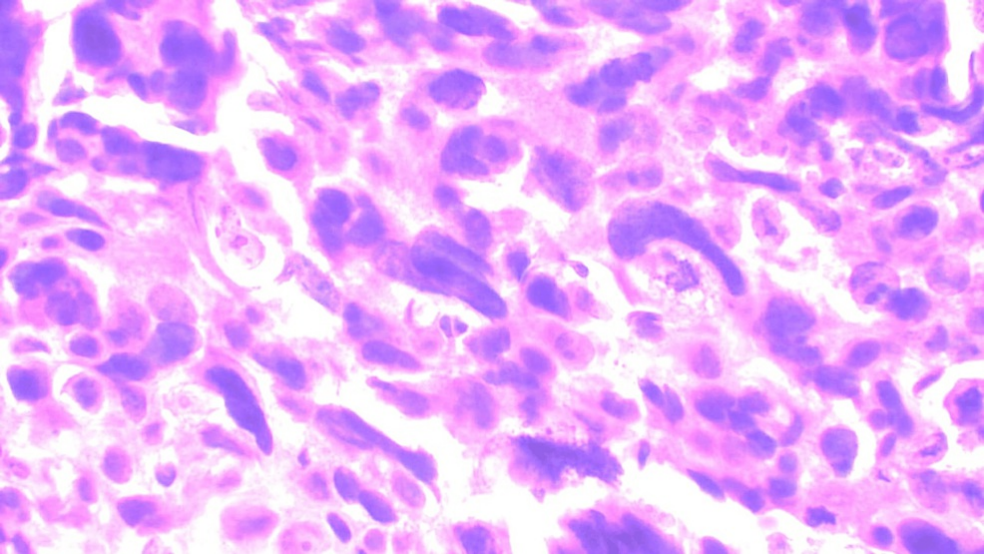
Hematoxylin and eosin staining of skin nodules over the right arm showing metastatic tumor cells.

An excisional biopsy was done from the skin nodule over the arm, which showed metastatic carcinoma deposits, which were positive for cytokeratin (Figure 4).

**Figure 5:**
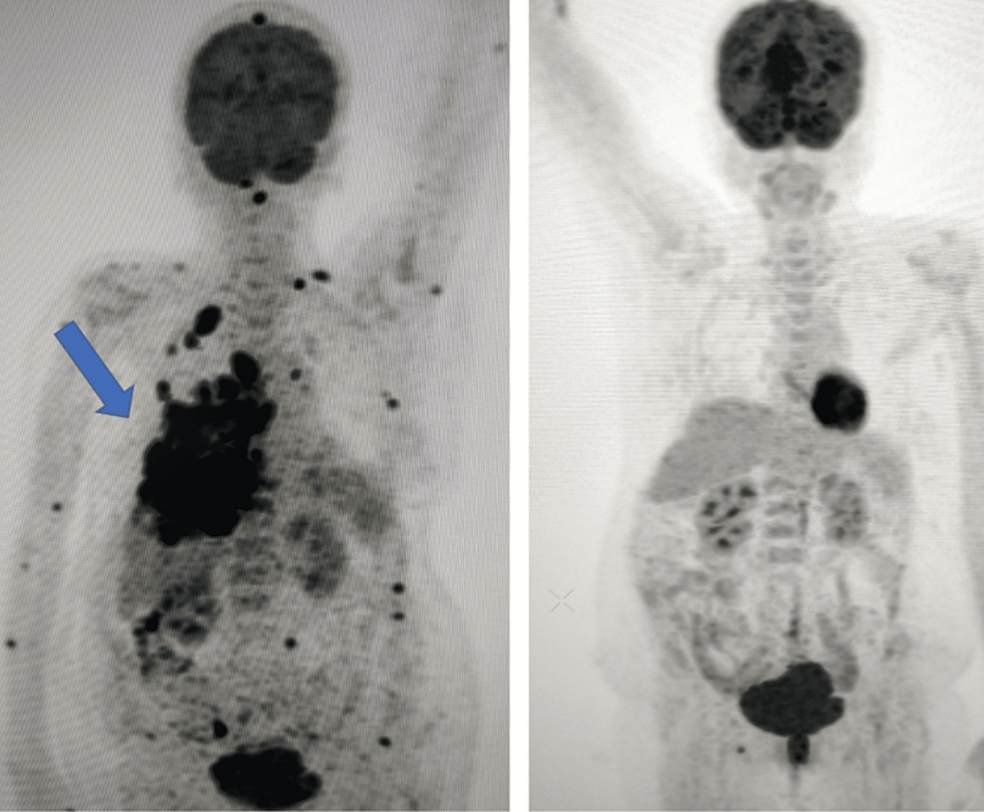
A comparison of baseline PET-CT scan (multiple skin and muscular lesions) with that after eight cycles of systemic therapy (complete metabolic response). PET-CT: positron emission tomography-computed tomography

18F-fluorodeoxyglucose positron emission tomography-computed tomography (18F-FDG PET-CT) scan was done for restaging. The findings were multiple FDG avid cutaneous deposits over the scalp, right arm, right forearm, and left arm. FDG avid muscular deposits were in the left sternocleidomastoid, left trapezius, left gluteus maximus, left paraspinal muscles, left adductor magnus, and left external oblique. Also, there were FDG avid right chest wall deposits along with multiple pleural deposits (Figure 5). The right sacrum also showed metabolically active lytic sclerotic lesions. Programmed death ligand 1 (PD-L1) expression was tested from the biopsy of the skin nodule. The tumor proportion score (TPS) was 0%.

She was then started with palliative chemotherapy injection bevacizumab 15 mg/kg, injection nano-dispersible paclitaxel 260 mg/m^2^, and injection carboplatin (area under the curve: 5), injection zoledronic acid 4 mg every three weeks, and tablet morphine for pain management. After three cycles of chemotherapy, there was a partial clinical response (a significant decrease in the size of subcutaneous nodules). So, the same regimen was continued for eight cycles.

A PET-CT evaluation was done after this, which suggested a complete metabolic response to therapy (Figure 5).

**Figure 6:**
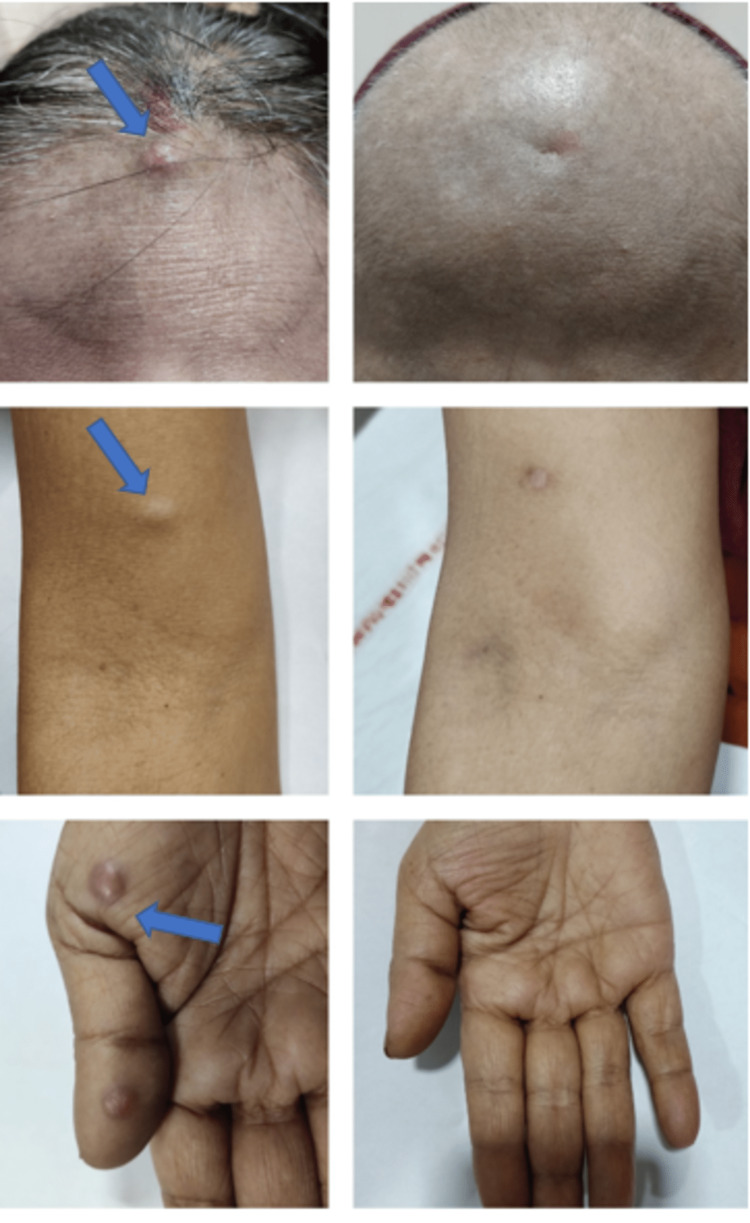
A comparison of the baseline skin nodules over the scalp, arm, and palm after systemic therapy.

Now, the patient is on maintenance injection bevacizumab 15 mg/kg, is doing well with an ECOG performance status of 1 with complete resolution of all skin nodules, and is pain-free (Figure 6). Thus, the duration from initial management to the last follow-up is 27 months, while the duration from recurrence is 12 months.

## Discussion

Metastasis from solid tumors generally occurs through three main modes: direct extension, hematogenous, and lymphatic. The most frequent sites of metastases in cervical cancer are the lung, liver, bone, and brain [4]. Skin metastasis from solid tumors is an uncommon finding. We hypothesize that the most plausible explanation for skin metastasis is through lymphatic channels. In females, skin metastases are in decreasing order from the breast, large intestine, melanoma, and ovary [5].

Cervical cancer with cutaneous metastasis is unusual. In a series of 1,190 patients with cervical cancer, only 15 (1.3%) were found to have skin metastasis during follow-up (biopsy, surgical specimen, or autopsy) [6].

The development of cutaneous metastasis varies with the stage of the disease. In stage I, the incidence was found to be 0.8%, and in stages II, III, and IV, it was 1.2%, 1.2%, and 4.8%, respectively [6]. In our case, the patient was of stage IIB. A review of case reports shows that most cases were of stage III, followed by stage II.

The definitive treatment of cervical cancer depends on multiple factors, such as the stage of the disease, associated comorbidities, the patient’s general condition, and the desire to preserve fertility. Among the reported cases of skin metastasis, four patients each underwent surgery and concurrent chemoradiotherapy, while five received EBRT alone. In a broad sense, it may be prudent to state that skin metastasis occurs irrespective of the modality of treatment chosen.

The histology of the disease contributes to the survival of the patient. Adenocarcinoma was more likely to die than squamous cell carcinoma [7]. It is unclear whether adenocarcinoma predicts a higher incidence of distant metastasis. However, a significant share of the reported skin metastasis cases is squamous cell carcinoma. This may reflect that more than 75% of cervical cancers are of squamous histology [8]. On the contrary, the incidence of skin metastasis is higher in adenocarcinoma compared to squamous cell carcinoma [6].

Skin metastasis from cervical cancer predisposes the lower abdomen and lower extremities more than the face, upper extremities, and chest. In this case, cutaneous metastasis occurred in the scalp and bilateral upper extremities, which is quite unusual. The presentation of cutaneous lesions was most often as non-tender nodules, which was the case with our patient.

The duration of development of skin metastasis after the treatment of cervical cancer varies widely. It will be noteworthy that this may occur before the completion of therapy [9]. In contrast, this sometimes takes more than a decade [10].

The cutaneous metastasis in cervical cancer is also associated with other visceral metastases, the lung being the most common.

The prognosis in these patients is generally dismal with a median survival of 12 months. So, the intention of management is palliation. Different treatment options include chemotherapy, biological response modifier, immunotherapy, EBRT, and electrochemotherapy [9]. The choice of therapy depends on multiple factors, such as the general condition of the patient, the presenting symptoms, the involvement of viscera, and the patient’s economic situation. The majority of patients received palliative chemotherapy. The commonly used chemotherapy was a combination of taxane and platinum.

The response to therapy is also highly variable, and the preferred imaging modality for response assessment is an 18F-FDG PET-CT scan. In the published literature, only a single case [11] showed a complete response to treatment. That patient presented with a localized plaque in the neck, and she underwent CCRT. The radiation dose was 50 Gray in 25 fractions, along with weekly injection paclitaxel.

On the other hand, our patient presented with florid cutaneous, muscular, and visceral metastasis with an ECOG performance status of 3. To the best of our knowledge, a combination of injection bevacizumab, injection carboplatin, and injection nano-dispersible paclitaxel was used for the first time in a case of skin metastasis. The outcome was encouraging. The patient not only tolerated the regimen well, but also, there was a dramatic improvement in performance status. This also translated to a complete metabolic response in the PET-CT scan.

Many of the case studies are silent regarding terminal events and survival. Among the rest of the reported literature, survival is less than six months after developing skin metastasis, thus cementing the fact that skin metastasis is a preterminal event.

A summary of case reports on skin metastasis that developed after the completion of treatment for cervical cancer is given in Table 1.

**Table 1:** Summary of case reports on cervical cancer with skin metastasis. HP: histopathology, CCRT: concurrent chemoradiotherapy, RT: radiotherapy, CT: chemotherapy, CR: complete response to therapy, PD: progressive disease, PR: partial response to therapy, BT: brachytherapy

Serial number	Author	Initial stage	Initial treatment	HP	Site of skin metastasis	Nature	Duration (months)	Other metastasis	Treatment	Response	Survival (months)
1	Benoulaid et al. [9]	IIIB	CCRT	Squamous	Lower abdomen, chest	Nodule	6	-	CT	PD	2
2	Benoulaid et al. [9]	IIIB	CCRT	Squamous	Arms, thigh, chest	Nodule	0	Lung, liver, bone	CT	PD	2
3	Khurana et al. [10]	IIIB	RT	Squamous	Thigh	Nodule	156	-	CCRT and interstitial BT	PR	-
4	Purbadi et al. [11]	IIA	Surgery	Adeno	Neck	Plaque	84	-	CCRT	CR	-
5	Katiyar et al. [12]	IIA	RT	Adeno squamous	Lower abdomen, back, thigh	Papule	60	Local	CT	PD	24
6	Cai et al. [13]	-	Surgery	Squamous	Face	Telangiectasia	24	Lung	CT	PR	-
7	Cherian et al. [14]	IVB	CT	Squamous	Breast, gluteal region, feet	Nodule	-	Lung	-	-	5
8	Basu et al. [15]	IIA	Surgery	Squamous	Upper thigh, lower abdomen	Nodule	12	-	CT/RT	PR	7
9	Elamurugan et al. [16]	IIIB	RT	Squamous	Palm, thigh	Nodule	10	-	-	-	-
10	Agrawal et al. [17]	IVA	CCRT	Adeno	Lower abdomen	Papule, plaque	2	-	CT	SD	6
11	Hayes et al. [18]	IIB	RT	Squamous	Back	Nodule	48	Liver, lung	-	-	1
12	Malfetano et al. [19]	IIIB	CCRT	Adeno	Lower abdomen	Nodule	1	-	CT	-	1
13	Malfetano et al. [19]	IIIB	RT	Squamous	Lower abdomen	Macule	4.5	Local	-	-	<1
14	Malfetano et al. [19]	IB	Surgery	Squamous	Lower abdomen	Macule	62	Local	-	-	1

## Conclusions

Cutaneous metastasis in cervical cancer is a rare clinical entity. The most common presentation is non-tender skin nodules. The common sites of occurrence are the lower abdomen and thigh. 18F-FDG PET-CT scan is a valuable modality for comprehensive response assessment. A combination of chemotherapy and bevacizumab can be considered in the management.
